# Genomic analysis of *Salmonella enterica* from cattle, beef and humans in the Greater Tamale Metropolis of Ghana

**DOI:** 10.1371/journal.pone.0325048

**Published:** 2025-06-18

**Authors:** Gabriel Temitope Sunmonu, Courage Kosi Setsoafia Saba, Erkison Ewomazino Odih, Opoku Bright, Eric Edem Y. Osei, Alfred Mensah, Saeed Abdallah, Abdul-Razak Alhassan, Stephen Wilson Kpordze, Olabisi C. Akinlabi, Anderson O. Oaikhena, Beverly Egyir, Iruka N. Okeke

**Affiliations:** 1 Department of Pharmaceutical Microbiology, Faculty of Pharmacy, University of Ibadan, Nigeria; 2 Department of Microbiology, Faculty of Biosciences, University for Development Studies, Tamale, Ghana; 3 Department of Biotechnology and Molecular Biology, Faculty of Biosciences, University for Development Studies, Tamale, Ghana; 4 Department of Bacteriology, Noguchi Memorial Institute for Medical Research, University of Ghana, Accra, Ghana; Federal University Oye-Ekiti, NIGERIA

## Abstract

*Salmonella enterica* is a bacterial foodborne pathogen that can infect humans and animals. Proper control of *Salmonella* requires routine surveillance and interventions across the food-production chain. However, due to limited resources the epidemiology and transmission of non-typhoidal *Salmonella* serotypes remain poorly understood in several African settings, including within Ghana. Here, we employed bacterial culture and whole genome sequencing (WGS) to investigate the prevalence, virulence and antimicrobial resistance determinants of *Salmonella enterica* isolates from beef, cattle blood and human patient stool in Greater Tamale Metropolis, Ghana. Enrichment and culture of the specimens yielded 62 isolates in total from beef (31), bovine blood (28) and human diarrhoeal specimens (3). We identified at least 15 STs and 18 different *Salmonella* serovars. The most frequently detected serovars were Poona (n = 13), Montevideo (n = 10) and Poano (n = 7) with *S.* Montevideo being the most common from cattle blood. Thirty-two (52%) isolates belonged to novel sequence types (STs), with ST2609 (n = 9) being most common. Four raw beef isolates harboured at least one gene conferring resistance to beta-lactam (*bla*_TEM-1_), chloramphenicol (*catA*), fosfomycin (*fosA7*), quinolone (*qnrD1*) or tetracycline (*tet(A)*). Eight isolates carried IncF, IncI and/or Col3M plasmid replicons. This study recovered *Salmonella*, often belonging to previously undocumented STs, at high frequencies from cattle and beef and demonstrated that isolates from human diarrhoeal patients are closely related to bovine isolates. The data highlight the need for broader and sustained surveillance and the urgent need for food safety interventions in Ghana.

## Introduction

*Salmonella* *enterica* is a well-known bacterial foodborne pathogen that causes a wide spectrum of infections in both humans and animals [1]. The enormous and global impact of *Salmonella* on public health and food safety stems from their aetiologic role in gastroenteritis and systemic infections, including bacteraemia [2,3]. Non-typhoidal *Salmonella* (NTS) infections, often zoonotic, are primarily caused by Subspecies 1 of *Salmonella enterica*, are often linked to contaminated animal products, such as poultry, eggs, and dairy, and typically result in gastrointestinal illness in humans [4]. While typically self-limiting, NTS can also lead to severe invasive infections in vulnerable sub-populations, such as young children, the elderly, and immunocompromised individuals, posing a substantial challenge to public health systems worldwide [5].

The incidence of *Salmonella* infections from meat and other animal products can vary depending on factors such as adherence to food safety regulations, prevalence in animals, handling, cooking methods, and epidemiological surveillance systems in place [6,7]. *Salmonella* contamination of animal products remains a significant concern for public health worldwide. In the United States, European Union member states, and many other parts of the world, regulatory agencies monitor and track foodborne illnesses, including those caused by *Salmonella*, through surveillance programs [8]. These programs help to estimate the incidence of *Salmonella* infections associated with beef consumption.

Despite the high incidence of both gastrointestinal and invasive non-typhoidal *Salmonella* infections [9,10], *Salmonella enterica* remains understudied across Africa due to inadequate clinical microbiology laboratory and surveillance infrastructure, as well as insufficient funding for existing systems [11,12]. Previously reported *Salmonella* serovars in Ghana include Kaapstad (ST4605 from beef and mutton), Hato (ST5308 from chicken; ST3899 from guinea fowl), Lagos (ST2469 from beef and goat) and Infantis (ST603 from goat) [13]. Additionally, *Salmonella* serovars identified in chicken eggs in Ghana include *S*. Enteritidis (ST11), *S*. Hader, and *S*. Chester. Strains of these serovars carry virulence genes, such as those encoding fimbrial adherence and secretion systems (*inv*, *spa*, *ssa* and *sse*), and resistance determinants against fluoroquinolones (*gyrA* (D87N), *qnrB*81), aminoglycosides (*aadA1*, *aph(3“)-Ib aph(6)-Id*), tetracycline (tet(A)), phenicols (*catA1*) and trimethoprim (*dfrA14 and dfrA1*) [14]. While some studies [13–16] have investigated the role of meat and other animal products in the spread of Salmonellosis in Ghana, no study has investigated its prevalence in slaughter cattle. Understanding the transmission dynamics and genetic diversity of *Salmonella enterica* across different reservoirs is crucial for devising effective control strategies and mitigating its associated risks. In this study, we determined the prevalence of *Salmonella enterica* in the beef value chain and among humans presenting with diarrhoea in hospitals in the Greater Tamale Metropolis of Ghana.

## Materials and methods

### Ethical considerations

Ethical approval for whole genome sequencing-based surveillance and research for this study was obtained from the Noguchi Memorial Institute for Medical Research, Ghana, NIMIMP-052/21/22.

### Experimental design and sample collection

Beef samples were randomly collected from vendors across the Greater Tamale Metropolis, while cattle blood samples were collected at the Greater Tamale Abattoir. The Tamale metropolis comprises north, south, east, west and central areas. An initial survey to identify vendors in these regions was done, and number codes allocated. Similarly, customers who came to slaughter their livestock at the abattoir were given number codes. Using random number generator from Microsoft excel, these codes were input and sampling codes for vendors and blood from cattle were obtained, respectively. These cattle are brought from a range of locations in Ghana, as well as from other countries, including Burkina Faso, Mali, and Niger, to the Greater Tamale Abattoir where they are slaughtered and processed for sale. Blood was collected at slaughter in collaboration with butchers to ensure that the animals experienced no additional harm beyond what is typical in standard butchering practices but no approval from an animal ethics committee was received at the time of collection. To reduce the chance of contamination, the first blood at slaughter was discarded after which 30–35 ml of cattle blood samples were collected aseptically into labelled sterile 50 ml tubes (Falcon®). Butchers and supervising laboratory scientists first sanitized their hands with 70% ethanol and then donned sterile gloves for each blood sample collection. This was repeated for each animal’s blood sampled. Faecal samples were collected from patients with diarrhoea attending the Tamale Central Hospital, Tamale West Hospital and Seventh Day Adventist Hospital between 31st October, 2016–21st May, 2017, using sterile stool containers. No clinical triage documentation is available. We collected and processed a total of 124 beef samples, 150 cattle blood samples, and 80 human stool samples. Stool samples were collected aseptically by patients under clinicians’ instructions using sterile stool-collection containers. All samples (beef, cattle blood and human faecal sample) were transported in ice chests with ice within 2 hrs of collection to the Spanish Laboratory Complex of the University for Development Studies, Tamale, Ghana for analyses for processing and isolation of bacteria. Coded bacterial isolates and meta-data for retrospective sequence generation and analysis in this study were processed in October 2022. The investigators did not have access to patient identifying information.

### *Salmonella* isolation and identification

*Salmonella enterica* were isolated on Xylose Deoxycholate (XLD) agar after pre-enrichment on peptone and selective enrichment on Modified Semi-Solid Rappaport-Vassiliadis (MSRV) agar. Bacterial colonies having morphological characteristics similar to *Salmonella* (a black centre and a slightly red coloured translucent zone) were sub-cultured to obtain pure colonies and Gram stained. PCR targeting the *invA* gene (284 bp) as described by Rahn et al. (1992) [17], using PCR oligonucleotides using PCR oligonucleotides *invA139f* GTGAAATTATCGCCACGTTCGGGCAA and *invA141r* TCATCGCACCGTCAAGGAACC as described previously [18]. PCR products were separated on 1.5% (w/v) agarose gels, stained with gel red (Biotium) and visualized using a transilluminator (UVP GelMax Imager).

Further biochemical profiling and biotyping of the isolates, was conducted using the Gram-negative (GN) test kit (Ref: 21341) on VITEK 2 systems (version 2.0, Marcy-l’Etoile, France, Biomérieux) according to manufacturer’s instructions. Strains identified as *Salmonella* biochemically, were whole genome sequenced.

### DNA extraction, library preparation and whole genome sequencing

Genomic DNA of the isolates was extracted using Wizard DNA extraction kit (Promega; Wisconsin, USA) in accordance with manufacturer’s protocols. The extracted DNA was quantified using a dsDNA Broad Range quantification assay on a Qubit fluorometer (Invitrogen; California, USA). DNA libraries were prepared using NEBNext Ultra II FS DNA library kit for Illumina with 96-unique indexes (New England Biolabs, Massachusetts, USA; Cat. No: E6609L). The DNA libraries were quantified using dsDNA High Sensitivity quantification assay on a Qubit fluorometer (Invitrogen; California, USA) and their average fragment length was determined using 2100 Bioanalyzer (Agilent). Libraries were sequenced on an Illumina MiSeq (Illumina, California, USA). The raw sequence reads were *de novo* assembled using SPAdes v3.15.3 [19] as implemented in the GHRU assembly pipeline (https://gitlab.com/cgps/ghru/pipelines/dsl2/pipelines/assembly). The details of the sequencing statistics showing sequencing depth, coverage and read length is presented in S1 Table and European Nucleotide Archive accessions (PRJEB58695) are in S2 Table.

### Sequence typing of *Salmonella* genomes

Sequence reads were deposited in the *Salmonella* database for *Salmonella* on EnteroBase v1.2.0 [20] to determine the multi-locus sequence types (MLST) using the Achtman 7 Gene scheme. The *Salmonella* genome assemblies were analysed using the *Salmonella* In-Silico Typing Resource (SISTR) for the prediction of serovars and serogroups [21] (https://github.com/phac-nml/sistr_cmd).

### Identification of plasmid replicons, antimicrobial resistance and virulence genes

Plasmid replicons present in the assembled genomes were detected using PlasmidFinder [22]. Antimicrobial resistance (AMR) genes carried by the isolates and the drug classes to which they possibly conferred resistance were predicted using AMRFinderPlus v3.10.24 [23] and its associated database (version 2022-04-04.1). We also detected the virulence genes present in the isolates using ARIBA [24] with the virulence factor database (VFDB).

### Phylogenetic analysis

We annotated the genomes of all *S. enterica* genomes using Prokka v1.14.6 [25] with default parameters and constructed a core genome alignment using Panaroo v1.3.2 [26] in ‘strict’ mode and excluding invalid genes. A core genome phylogeny was then inferred from the resulting alignment using RaxML-NG v1.1.0 [27] with the generalised time reversible gamma model, 20 random and parsimony-based starting trees, and 1000 bootstrap replicates with convergence estimation every 50 iterations. Bootstrapping converged after 700 replicates. To determine the degree of phylogenetic relatedness between the strains, reference-based phylogenies were also constructed for each serovar with at least five genomes. Complete representative reference sequences for each serovar were systematically selected from the National Center for Biotechnology Information Reference Sequence (RefSeq) database (https://www.ncbi.nlm.nih.gov/refseq/) using BactinspectorMax v0.1.3 (https://gitlab.com/antunderwood/bactinspector). Accession numbers for the selected references include GCF_000486765.2 (Poano), GCF_900478385.1 (Poona), and GCF_003031875.1 (Montevideo). For each serovar, the sequence reads were then mapped to the chromosome of the respective reference genome using BWA (v0.7.17) [28] and variants were called and filtered using BCFtools v1.9 [29] as implemented in the GHRU single nucleotide polymorphism (SNP) phylogeny pipeline (https://gitlab.com/cgps/ghru/pipelines/snp_phylogeny). Variant positions were concatenated into a pseudoalignment and used to generate a maximum likelihood tree using IQ-TREE v1.6.8 [30]. SNP distances between the genome pairs were calculated using snp-dists v.0.8.2 (https://github.com/tseemann/snp-dists).

## Results

### Prevalence of *Salmonella* in the studied specimens

Culture and initial tests on MSRV and XLD yielded presumptive *Salmonella* from 37/124 (29.8%) raw beef samples, of which 31 (25% of the 124 specimens) were eventually confirmed as *S. enterica* based on whole genome sequencing (WGS). For the cattle blood samples, 37/150 (24.7%) were positive for presumptive *Salmonella*, and 28/150 (18.7%) were confirmed positive. Among the human faecal samples, recorded 3/80 (3.8%) were positive for presumptive *Salmonella,* all of which were confirmed to be *S. enterica* based WGS. In all, the genomes of sixty-two *Salmonella* isolates were sequenced in this study; 31 were from raw beef, 28 from blood of slaughtered cattle, and three from human stool.

### *Salmonella* serotypes, sequence types, and phylogeny

The most common serotypes detected in this study were Poona (n = 13, 21%), Montevideo (n = 10, 16.1%), Poano (n = 7, 11.3%), Chester (n = 5, 8.1%) and Give (n = 4, 6.5%). Other represented serotypes include; Gaminara (n = 3), Llandoff (n = 3), Muenster (n = 3), Odozi (n = 3), Bredeney (n = 1), Labadi (n = 1), Pisa (n = 1), Scarborough (n = 1), Vinohrady (n = 1), Yoruba (n = 1), I 1,3,19:z:- (n = 2), I 28:g,t:- (n = 2) and I 4:y:- (n = 1) (S3 Table).

**Fig 1:**
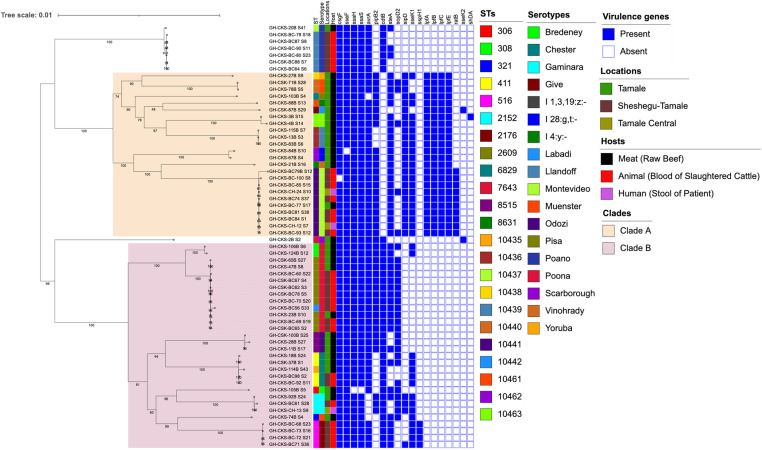
Core genome phylogeny of *Salmonella* isolates. A mid-point rooted maximum likelihood phylogenetic tree based on core genome and created using IQ-Tree annotated with ST, serotypes, location of isolation, host of isolates and virulence genes not shared by all isolated in the assembled genomes. The data are available in interactive format on Microreact (https://microreact.org/project/frWNVcb5zLZy22GfXAbBnL-ghru-ghcks2006-salmonella-enterica).

The most common sequence types (STs) were ST2609 and ST411 which were represented by ten (14.5%) and four (6.5%) isolates respectively (Fig 1). As many as 32 (51.6%), including all 10 Montevideo (ST10441) and seven isolates of the rare Poano serotype (ST10439 (n = 6), ST10437 (n = 1)) belonged to a novel ST (S4 Table). The 13 *S*. Poona isolates belonged to ST2609 (n = 10), ST308 (n = 2), and novel ST10442 [1]. Four of the Chester serotype strains belong to ST411 while one is a single locus variant of the sequence type (ST411*). The four Give serotype belong to ST516 (Fig 1, S3 Table).

The most serotype and sequence type diversity in this study was seen among raw beef and slaughtered cattle blood isolates. Uncommonly recovered ST306 (n = 1), ST308 (n = 2), ST321 (n = 1), ST2176 (n = 1), ST7643 (n = 1), ST8515 (n = 2) and ST8631 (n = 1) were found only in raw beef isolates and ST516 (n = 4) was found only in blood of slaughtered cattle. Two of the human stool isolates belong to Montevideo serotype and a previously unreported sequence type (ST10441) while the third belongs to ST2152 and the Gaminara serotype (Fig 1, S3 Table). Both of these serotypes and STs were also recovered from bovine sources in the study.

A core genome-based phylogeny of 62 *Salmonella* isolates from this study is presented in Fig 1. Isolates belonging to the same serovar clustered together on the phylogenetic tree, except the three *S.* Muenster strains. Two of the *S.* Muenster strains, belonging to a Novel ST (ST10440), clustered together and away from the third (ST321) strain, which was located on a distinct branch on the tree and most closely related to the four *S.* Give strains. To determine the phylogenetic relatedness between isolates belonging to the same serovar, we reconstructed whole genome reference-based phylogenies for each distinct serovar with ≥5 genomes (S1 Fig). The two human *S*. Montevideo isolates were identical (0 SNPs) to six other *S*. Montevideo isolates from cattle blood (n = 5) and meat (n = 1). The two remaining animal *S*. Montevideo isolates differed by 1–2 SNPs from the rest of the *S*. Montevideo strains (S5 Table). Conversely, the *S.* Poano isolate from beef was distantly related (≥600 SNPs) to the six isolates from cattle blood, which were almost identical (≤1 SNP) (S6 Table). Among the *S.* Poona isolates, one beef isolate was identical to seven isolates from cattle blood (≤1 SNP) (S7 Table).

### Virulence factors, plasmid replicons and antimicrobial resistance gene profiles of *Salmonella*

All the isolates carry multiple genes that have been shown to be important for *Salmonella* virulence. The curli (*csg*) genes were present in all *Salmonella* isolates as well as the fimbrial operon *bcf*, and *fim*) genes. All the isolates had at least two *ste* fimbrial genes, *steB* and *steC*. Long polar fimbriae (*lpf*) genes were present in isolates from all serovars except *S.* Bredeney, Chester, Gaminara, Give, Odozi, Poano, Poona, and Scarborough, accounting for 38.7% (24/62) of the isolates. Only the *S*. Montevideo strains carried the virulence factor *ratB,* associated with long-term intestinal persistence and the I 1,3,19:z:- strain carry *shdA,* which encodes an autotransporter. The *inv*, *org*, *prg*, *sif*, *spa*, *ssa*, *ssc*, *sse*, *ssp* and *sop* type III secretion system effector genes were detected in all the isolates while *avr* was present in 91.9% (57/62) of the isolates. Thirty-eight (61.3%) of the isolates also carry the cytholethal distending toxin gene, *cdtB*. Genes involved in host cell manipulation and invasion such as: *slrP*, *sptP*, *sicP*, *sicA*, *pipB*, *mig-14*, *misL*, *sinH* and *sseL* were present in all isolates while *pipB2* was present in 66.1% (41/62) of isolates (Fig 1, S3 Table). The virulence genes *gogB* and *grvA* were absent in all strains.

Most of the *Salmonella enterica* isolates (58/62) harbour no AMR genes. However, four isolates from raw beef carry at least one of the gene conferring resistance to beta-lactam (*bla*_TEM-1_), chloramphenicol (*catA*), fosfomycin (*fosA7*), quinolone (*qnrD1*) and tetracycline (*tet(A)*) (S3 Table). The plasmid replicons identified in this study were IncFII(S) (n = 4), IncFII(SARC14) (n = 2), IncFII (n = 1), IncFIB(AP001918) (n = 1), IncI2(Delta) (n = 1), Col3M (n = 1) and IncI1-I(Gamma) (n = 1). The plasmid replicons were spread across eight isolates (6 raw beef and 2 blood of slaughtered cattle isolates). Four of the ST2609 serovar Poona isolates harbour one of either IncFII(S) or IncFII(SARC14) while one raw beef isolate (GH-CKS-124B_S12) harbour three plasmid replicons (IncFII(SARC14), IncFII and IncI2(Delta)) (S3 Table).

## Discussion

In this study, we recovered *Salmonella enterica* isolates from beef, cattle blood and, to a lesser extent, from human stools. We employed a robust pre-enrichment (with peptone), enrichment (on MSRV agar) and selection (on XLD agar) protocol however it is possible that the use of multiple enrichment media, e.g., selenite might have recovered other Salmonellae. In spite of this limitation, 31 (25%) of beef samples yielded a *Salmonella* isolate and considerable diversity was seen – at least 15 STs and 18 different *Salmonella* serovars recovered from cattle blood, raw beef and human stool from this study. Most of these serovars and STs have not been previously documented in Ghana, highlighting the need for more precise surveillance through whole genome sequencing, which contributes to our understanding of *Salmonella* serovar diversity in Ghana and underscores the importance of continued monitoring to inform targeted public health interventions.

A few serovars that are globally disseminated and highly prevalent worldwide were detected in this study, including Montevideo, Muenster and Poona but most of the serovarieties detected in the present study were uncommon in the literature overall (e.g., *S.* Poano and Odozi) or outside African settings (e.g., *S.* Yoruba) [16,31–34]. Recovery of serovars like *S.* Give and *S.* Yoruba, adds to data that suggests that these are endemic in Africa. A number of STs belonging to these and more commonly reported serovars were novel. With the exception of antimicrobial-resistant *S.* Poona isolated from poultry meat [35] and humans [36], and *S.* Give isolated from milk and associated utensils [16], most of the *Salmonella* serovars identified in this study have not yet been reported in Ghana. *S.* Gaminara has been associated with citrus and other fruits [37–40] and water [41,42] whereas in this study, one human stool and raw beef isolate was identified as *S.* Gaminara. Isolation of *S.* Montevideo has also been reported in buffalo meat in Egypt [43], in both cattle and human in the US [44], in non-diarrhoeic dogs in Grenada, West Indies [45] and in dairy farms in Uruguay [46]. Similarly, in this study *S.* Montevideo was present in human stool, raw beef and blood of slaughter cattle. Interestingly, with the exception of *S.* Give serovars of 14 isolates recently reported from food-preparation sources in Northern Ghana (Fresno, Give, Orleans, Infantis, Agona and Plymouth) [16] were not seen in this study. S. Give again, was the only one of the 14 serovars from this or the Sunmonu et al study that was recently reported from the poultry food chain in Nigeria [47]. Detection of uncommon serovars, the limited overlap in serovar type in different studies and the wide variety of serovars overall even with small sample sizes highlight a surveillance gap for foodborne *Salmonella* in Ghana and elsewhere in West Africa, and points to the need for further and larger epidemiological studies to identify serovars and lineages of regional significance. The identification of these serovars in various sources further underscores the importance of understanding reservoirs and transmission pathways within Ghana.

Some of the serovars identified in this study have been previously implicated in human infections. *S.* Montevideo has been associated with bacteraemia [48] and linked to foodborne illness outbreaks involving contaminated vegetables and spices [49]. Similarly, *S.* Poona has been reported to cause bacteraemia [50] and has been involved in foodborne outbreaks from cucumbers [51]. *S.* Give has been connected to an outbreak of gastroenteritis in Germany, which involved 115 cases linked to the consumption of raw minced pork [52]. Additionally, *S.* Give has been reported to cause brain abscess and bacteraemia in India [53]. In the US, multistate outbreak of *S.* Chester infections was associated with frozen meals, affecting 44 people [54]. *S.* Muenster has been isolated from human blood and livestock in the same household in Burkina Faso [55] and it has also been found in humans with clinical diarrhoea in Egypt [56]. *S.* Bredeney has been implicated in cases of bacteraemia in Cyprus [57]. The presence of these serovars in cattle meat and blood in Ghana, further highlights their potential to cause human infections.

A very concerning finding in this study is that *Salmonella* belonging to serovars Gaminara and Montevideo were recovered from three of 80 human stool specimens and that all three isolates were closely related to bovine isolates. *S.* Montevideo isolates were also the most common serovar of slaughter cattle blood isolates. The data in this suggest possible interchange of *Salmonella* between humans and livestock/meat but the limited number of human isolates (n = 3) restricts conclusions about transmission between livestock/meat and humans.

The IncFII(S) plasmid replicon was detected more commonly than any other in *Salmonella* genomes from this study. Plasmids with this replicon have been reported beta-lactam [58–61], tetracycline [58,61] and aminoglycoside [58] resistance, as well as virulence [58,59] in *Salmonella*. Other plasmid replicons detected in this study such as: IncFII(SARC14) [62], IncFII [61,63], IncFIB(AP001918) [61], IncI2(Delta), Col3M [64] and IncI1-I(Gamma) [65] have been reported to carry AMR genes in *Salmonella*. However, in this study none of the isolates carrying IncFII(S) and IncFII(SARC14) replicons harbour any resistance genes. This may reflect a lack of selective pressure in Ghana’s beef production sector. However, the presence of replicons associated with resistance means that platforms are in place for rapid evolution to resistance. The *S.* Bredeney ST306 isolate harbouring the plasmid-mediated quinolone resistance gene *qnrD1* carries Col3M and IncI1-I(Gamma) plasmid replicons and one of the S. Poona ST308 isolates (GH-CKS-124B_S12) carrying IncFII, IncFIB(AP001918) and IncI2(Delta) also harbours tetracycline (*tet(A)*) and beta-lactamase (*bla*_TEM-1_) resistance genes. IncI2 plasmids are commonly reported from *E. coli,* where they carry multiple AMR genes [66], but rarely present in *Salmonella* and when detected, do not usually harbour any resistance determinants [46,67]. In this study, we found an IncI2 replicons in a *Salmonella* Poona isolate that carried resistance genes but also two other replicons. As long-read sequencing was not conducted in this study, it is not possible to fully understand the nature of the genetic elements carried by this and other strains as well as to decipher which resistance genes are on which replicon.

Not all recent studies conducted along the beef food chain in Ghana have recovered *Salmonella* [68], but most have [13,15,16,69,70] and therefore preventive actions need to be stepped up. These should include improved hygiene and sanitation practices, strengthened surveillance and monitoring systems, stricter food safety standards and regulations, vaccination and treatment programs for cattle, and public health awareness campaigns.

## Conclusion

This study has identified a range of *Salmonella* serovars in slaughter cattle and beef in Ghana. The serovarieties and virulence gene repertoires of the recovered strains suggest pathogenic potential and recovery of strains belonging to two lineages identified from humans visiting health centres in the region further gives cause for concern. While antimicrobial resistance was not common in this study, resistance genes and mobile elements capable of carrying resistance genes were identified in many of the isolates, emphasizing the need to avoid transmission of *Salmonella* across One Health boundaries in Ghana. As a first step to effectively combat the spread of *Salmonella*, it is essential to foster collaboration among various stakeholders in the One Health framework to boost surveillance, including public health officials, veterinarians, food safety experts, and environmental scientists, thus creating a comprehensive and integrated approach to AMR surveillance, and then control.

## Supporting information

S1 Fig Reference-based phylogeny of *Salmonella* serovars with at least five genomes. (DOCX)

S1 Table Sequencing statistics. (CSV)

S2 Table Accession IDs. (CSV)

S3 Table Isolates’ metadata including plasmid replicon, AMR and virulence profile. (CSV)

S4 Table Isolate STs and allelic profiles. (CSV)

S5 Table *S.* Montevideo SNP distance matrix. (CSV)

S6 Table *S.* Poano SNP distance matrix. (CSV)

S7 Table *S.* Poona SNP distance matrix. (CSV)
