# Supplementary material for: Genomic analysis of Salmonella enterica from cattle, beef and humans in the Greater Tamale Metropolis of Ghana
Source: PLoS One. 2025 Jun 18;20(6):e0325048. doi: 10.1371/journal.pone.0325048 (PMC12176171; doi:10.1371/journal.pone.0325048)
Supplement: S1 Fig — (DOCX) [file pone.0325048.s001.docx]

Supplementary Figure 1: Reference-based phylogeny of *Salmonella* serovars with at least five genomes. A. Montevideo (<https://microreact.org/project/dr2skNEtp4jAzGDv1xqnYt-ghru-ghcks2006-salmonella-enterica-montevideo>) B. Poano (<https://microreact.org/project/5XNG1yZeDzsBch9iCpqmPu-ghru-ghcks2006-salmonella-enterica-poano>) C. Poona (<https://microreact.org/project/osJ8FBdALtGDPgdVct8cBG-ghru-ghcks2006-salmonella-enterica-poona>). A mid-point rooted maximum likelihood phylogenetic tree based on SNP alignment and created using IQ-Tree annotated with ST, serotypes, location of isolation, host of isolates.
